# Optimizing Short-Term Maximal Exercise Performance: The Superior Efficacy of a 6 mg/kg Caffeine Dose over 3 or 9 mg/kg in Young Female Team-Sports Athletes

**DOI:** 10.3390/nu16050640

**Published:** 2024-02-25

**Authors:** Houda Bougrine, Achraf Ammar, Atef Salem, Khaled Trabelsi, Haitham Jahrami, Hamdi Chtourou, Nizar Souissi

**Affiliations:** 1 High Institute of Sport and Physical Education Gafsa, University of Gafsa, Gafsa 2100, Tunisia; houdabougrine@live.fr; 2 Physical Activity Research Unit, Sport and Health (UR18JS01), National Observatory of Sports, Tunis 1003, Tunisian_souissi@yahoo.fr (N.S.); 3 High Institute of Sport and Physical Education of Sfax, University of Sfax, Sfax 3000, Tunisia; trabelsikhaled@gmail.com; 4 Department of Training and Movement Science, Institute of Sport Science, Johannes Gutenberg-University Mainz, 55099 Mainz, Germany; 5 Research Laboratory, Molecular Bases of Human Pathology, LR19ES13, Faculty of Medicine of Sfax, University of Sfax, Sfax 3029, Tunisia; 6 Interdisciplinary Laboratory in Neurosciences, Physiology and Psychology: Physical Activity, Health and Learning (LINP2), UFR STAPS (Faculty of Sport Sciences), Université Paris Lumières (UPL), Paris Nanterre University, 92000 Nanterre, France; 7 Research Laboratory, Education, Motricity, Sport and Health (EM2S), LR15JS01, High Institute of Sport and Physical Education of Sfax, University of Sfax, Sfax 3000, Tunisia; 8 Department of Psychiatry, College of Medicine and Medical Sciences, Arabian Gulf University, Manama 323, Bahrain; hjahrami@health.gov.bh; 9 Ministry of Health, Manama 410, Bahrain; 10 High Institute of Sport and Physical Education Ksar-Saïd, Manouba University, Mannouba 2010, Tunisia

**Keywords:** caffeine intake, dosages, mild consumers, side effects, female athletes, team sports, athletic performance

## Abstract

Caffeine (CAF) is among the most extensively researched dietary supplements worldwide. However, little is known about the relationship between dosage and performance enhancement, particularly in female athletes. This study aimed to explore the effects of three different CAF dosages (3 mg·kg^−1^, 6 mg·kg^−1^, and 9 mg·kg^−1^) on high-intensity exercise and the prevalence of undesirable side effects related to these doses among female team-sports athletes. All participants (n = 16; age: 16.9 ± 0.6 y; height: 1.64 ± 0.1 m; BMI: 21.6 ± 1.5 kg·m^−2^) were mild CAF consumers. This study had a randomized, crossover, double-blind design in which each athlete performed four experimental sessions after ingesting either a placebo (PLAC), 3 mg·kg^−1^ CAF (CAF-3), 6 mg·kg^−1^ CAF (CAF-6), or 9 mg·kg^−1^ of CAF (CAF-9), with an in-between washout period of at least 72 h. In each experimental session, 60 min after ingesting the capsules, participants underwent a countermovement jumps test (CMJ), modified agility t-test (MATT), repeated sprint ability (RSA) test, and a rating of perceived exertion (RPE) and completed the CAF side effects questionnaire. Our findings revealed that in comparison to the PLAC condition, the MATT, RSA_mean_, and RSA_best_ performances were significantly greater only under the CAF-6 and CAF-9 conditions. Although the RPE scores remained unchanged, CMJ performance improved under all CAF conditions. All the performance outcomes were better for the CAF-6 and CAF-9 conditions than for the CAF-3 condition. Notably, no significant difference between the CAF-6 and CAF-9 conditions was observed for any of these parameters despite the highest incidence of side effects being noted for the CAF-9 condition. In summary, our findings highlight the recommendation for a moderate CAF dosage of 6 mg·kg^−1^ rather than 3 or 9 mg·kg^−1^ to enhance various aspects of short-term maximal performance in mild-CAF-consumer female team-sports athletes while mitigating the occurrence of adverse CAF side effects.

## 1. Introduction

Caffeine (CAF) is a key ingredient in coffee and is the most popular drink after water. It is estimated that people around the world consume more than 2 billion cups of this beverage every day [1]. Despite its lack of nutritional value and nonessential role in biological functions, CAF (1,3,7-trymethylxantine), a pharmacologically active substance, is exceptionally popular, as it is present in a variety of foods, beverages, and nutritional supplements and is estimated to be consumed daily by approximately 80% of the world’s population [2,3]. 

CAF consumption among athletes, particularly in endurance sports, has significantly increased since its removal from the World Anti-Doping Agency’s prohibited substances list in 2004, with usage trends rising with age [4,5]. CAF is widely recognized as one of the most commonly utilized ergogenic aids in sports, and numerous studies have validated its effectiveness in enhancing both anaerobic and aerobic performance [6]. Due to its lipophilic nature, CAF is quickly taken into the body after it is ingested orally, and it is capable of crossing all biological barriers, including the blood–brain barrier [7]. This psychoactive substance is proposed to enhance performance through various potential mechanisms, which include preserving muscle glycogen by inhibiting phosphodiesterase [8,9], promoting calcium release from the sarcoplasmic reticulum [10,11], and counteracting the effects of adenosine A1 and A2 receptors in the central nervous system [12]. Therefore, it is plausible that either a single factor or an amalgamation of these factors might contribute to the enhancement in exercise performance following CAF intake. The efficacy of CAF as a performance enhancer depends on variables such as dosage, form, training status, timing of consumption, habitual CAF intake, sex, and exercise type [13]. Moreover, recent studies suggest that genetic variations in the CYP1A2 and ADORA2A genes influence the impact of CAF on exercise performance [6,14]. This calls for more extensive investigations and a greater understanding of the appropriateness of CAF in different scenarios. Although CAF supplementation may improve performance in individual sports, its effectiveness is less evident in team sports, where success is determined by a combination of physical condition, technical skill, and tactical understanding [15]. Research on this topic, however, has been inconclusive; recent meta-analyses findings have shown that CAF can be effective for jumping performance [16,17], agility performance [18], and repeated sprint ability [16,17], while others have indicated that it has no effect on jumping and agility performance [19] or repeated sprint bouts [20]. These contradictory results could be due to the different dosages of CAF used in various studies, an aspect that requires additional attention within athletic groups.

On the other hand, recent data show a predominant focus on male participants, with male-only samples ranging from 72% to 100% across the 21 meta-analyses from an umbrella review [13]. Current CAF supplementation guidelines, primarily based on studies involving male athletes, are identically applicable to females despite a lack of research specifically analyzing female athletes, which raises practical concerns [6]. In this context, recent findings underline the need for sex-specific guidelines in athletics, highlighting that conclusions drawn from observations in male groups may not be applicable to female groups due to potential sex differences [21].

In female team-sports athletes, lower doses, namely between 1 and 3 mg·kg^−1^, have been found to have little to no effect on aspects such as jumping [22,23], agility [22,23,24], or repeated sprint [22,23,25]. However, interestingly, the same dosage was shown to have beneficial effects on jumping [26] and agility [26]. On the other hand, a moderate dose of 6 mg·kg^−1^ did not have any noticeable effect on jumping [27,28], agility [29,30], or repeated sprint ability [30,31]. However, contradictory effects were reported in other studies in which the same dose was found to improve jumping performance [30,32,33], agility [32], and repeated sprinting [33]. Recent findings indicated that (5 mg·kg^−1^) of CAF intake has been linked to improved CMJ and grip strength among female volleyball players [34]. These results highlight the varied responses to CAF intake in the context of female sports performance, suggesting a need for further research. Investigations into various CAF effects on female team-sport athletes have proven to be insightful but also expose a deficiency in existing data. Karayigit et al. [1] showed that consuming low (3 mg·kg^−1^) and moderate (6 mg·kg^−1^) doses of CAF in the form of coffee may equally enhance lower body muscular endurance. Meanwhile, another study conducted by Arazi et al. [35] indicated that ingesting a higher (5 mg·kg^−1^) dose of CAF may reduce pain perception during muscular endurance tests in female karate athletes, an effect not observed with smaller doses of 2 mg·kg^−1^. Furthermore, Karayigit et al. [36] illustrated that low (3 mg·kg^−1^) and moderate (6 mg·kg^−1^) doses of CAF consumption could increase the average peak power score during repeated sprints test on a cycle ergometer on trained female team-sports players. Despite these insights, the difference in outcomes between lower, moderate, and high doses of CAF on critical aspects of the performance of high-intensity exercise in team sports, such as vertical jump, agility, and repeated sprints, are not well documented, presenting a fertile opportunity for further exploration.

To explain this divergence, the data around CAF dosages of 3–6 mg·kg^−1^ are inconclusive, suggesting that not everyone benefits from these levels and that future studies should consider that lower CAF doses (≤3 mg·kg^−1^) primarily affect the CNS, while higher doses (6–9 mg·kg^−1^) may have peripheral effects [8,37,38]. The impact of increased CAF consumption should be investigated in relation to individual tolerance and sensitivity to CAF. Thus, further investigations involving the administration of higher doses of CAF (i.e., 6–9 mg·kg^−1^), as opposed to the use of lower doses of CAF (≤3 mg·kg^−1^), should be carried out [37]. CAF has been shown to enhance exercise performance across various dosage ranges (2–9 mg·kg^−1^), although the physiological processes facilitating these improvements at higher doses have not been determined [37]. A more effective methodology for studying CAF effects might involve conducting trials with both low and high CAF doses within each study, thus enabling a direct comparison. Despite the apparent logic of this approach, its implementation in research remains surprisingly rare [39]. However, further studies are needed to determine whether a linear relationship exists between dosage and performance enhancement. Interestingly, studies have not thoroughly investigated the impact of CAF on women at either high doses exceeding 9 mg·kg^−1^ or at extremely low doses [17]. This understanding could significantly contribute to the interpretation of the physiological processes associated with CAF effectiveness, particularly in female athletes. Moreover, the increase of the CAF dose should be based on the individual’s response to the substance, the habitual CAF intake classification (low, mild, moderate, or heavy), the type of physical exercise, sex, and the prevalence of side effects after pre-exercise CAF intake. Surprisingly, studies exploring the impacts of CAF withdrawal on exercise performance are lacking, indicating a pressing need for research in this area to develop an effective restriction protocol. Despite the positive impact of CAF on physical factors, some argue that team-sport athletes should carefully use CAF due to its potential impact on the technical and tactical aspects, with associated side effects like nervousness possibly leading to a decline in accuracy performance [16]. However, the potential negative side effects of CAF consumption, particularly at the levels taken to enhance performance, have received relatively little focus [40].

Furthermore, the trend of early specialization in sports seems to be on the rise among young athletes [41,42], with the pressure to focus on one sport coming from coaches, parents, and peers [43]. This may lead young athletes to start using supplements at a young age without specific recommendations for their age group, potentially leading to adverse effects for some athletes. In this context, limited studies have explored CAF’s impact on young athletes, resulting in scarce evidence that draws definitive conclusions or guidelines. The performance advantages seen in adults may not extend to younger individuals, highlighting the need for additional research in the youth athlete demographic [16]. Limited data are available regarding the effects of CAF on young female athletes. Therefore, our study seeks to investigate the impact of CAF intake on this specific demographic.

In light of the above considerations, the objective of this study was to examine the effects of three distinct dosages of CAF (3 mg·kg^−1^, 6 mg·kg^−1^, and 9 mg·kg^−1^) on high-intensity physical performance and the prevalence and severity of any possible side effects associated with these dosages among young female team-sports athletes.

## 2. Materials and Methods

### 2.1. Participants

Following the recommended guidelines proposed by Beck [44], we used G*Power software (version 3.1.9.6; Kiel University, Kiel, Germany) [45] to pre-determine the required sample size. The significance level (α) was established at 0.05, with a desired statistical power (β) of 0.95. Based on Karayigit et al. [1] and discussed between authors, we approximated the effect size to be 0.5. To attain the requisite statistical power, it was determined that a sample size of at least 10 athletes would be sufficient, therefore minimizing the probability of a type 2 statistical error.

Among the 39 reviewed surveys, 25 females team-sports players were considered suitable and volunteered to participate in the study. However, during the experimental phase, nine participants withdrew due to logistical (one player) and menstrual cycle (eight players) reasons. The data gathered from the 16 participants who successfully completed all the sessions of the experiment are shown in Table 1. Before providing their written consent, the athletes and their parents were briefed about various aspects of the experiment, including the schedule, the type of exercise, and the evaluations they would be required to undergo. All protocols and methods received approval from the local research ethics committee of the University of Jendouba (054-2023), adhering to the latest version of the Declaration of Helsinki.

The study included young female team-sports athletes (well-trained handball (n = 10) and football (n = 6) players) who volunteered to participate in this investigation (Table 1). Participants were selected based on the following inclusion criteria: (1) aged between 15 and 20 years; (2) actively participated in team sports for at least 3 years, with a minimum of 3 times/week for the last 6 months; (3) had daily CAF intake less than 2.99 mg·kg.day^−1^; and (4) had regular menstrual cycles with a variance of no more than 3 days over the last 4 months [46].

The individuals who were excluded from the study met the following exclusion criteria: (1) had a history of a menstrual disorder within the past four months; (2) had taken oral contraceptives or medications in the previous four months, including pills, patches, injections, implants, and intrauterine devices; (3) had a positive alcohol and/or smoking status; (4) had a history of diseases and/or the use of any medications for any chronic medical condition; (5) had experienced an injury within the last 3 months; (6) had taken stimulants, narcotics, mind-altering drugs, nutritional enhancers, or participated in any strict dietary regimen during the previous four months that could influence hormone balances or sporting performance; (7) had extreme chronotypes; (8) were allergic to CAF; and (9) had PSQI scores more than 5.

Player chronotypes for this study were identified through Horne and Ostberg (1976) self-assessment questionnaire [47]. This questionnaire measures preferences for sleep and activity using 19 items on a Likert scale and was used to avoid circadian typology influencing the study results. Participants with extreme morning or evening tendencies were excluded. The selected study participants were those with “neither type” chronotype. According to the Pittsburgh Sleep Quality Index (PSQI), all the selected athletes had sleep durations of approximately 7.7 ± 0.6 h during the month leading up to the experimental procedure [48]. All the athletes had PSQI scores less than 5, indicating good sleep quality [49]. Furthermore, the study recruited participants who were all classified as mild CAF consumers (1.09 ± 0.3 mg·kg·day^−1^), based on the recent classification proposed by Filip et al. [50]. This classification was introduced to standardize the categorization of athletes, aiming to minimize the probability of disparities in the qualification of daily CAF intake across various studies. These data were assessed during the four weeks before the commencement of our experiment by a reliable semi-quantitative self-reported CAF intake questionnaire [51]. Using a mobile application, Mycalendar^®^ Period Tracker, which tracks key events throughout the menstrual cycle, each participant was evaluated during the follicular phase, the luteal phase, or both phases of their menstrual cycle [52]. However, recent investigations suggest that the bodily absorption and effects of acute caffeine consumption are constant across different menstrual phases, and female athletes consistently gain from their intake [53,54].

### 2.2. Experimental Design

**Figure 1:**
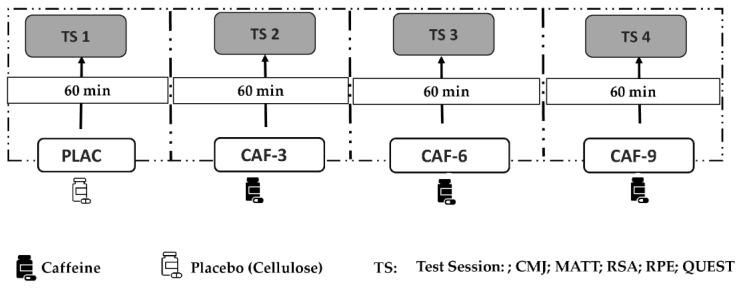
Study design: PLAC, placebo; CAF, caffeine; CAF-3, 3 mg·kg^−1^ of CAF; CAF-6, 6 mg·kg^−1^ of CAF; CAF-9, 9 mg·kg^−1^ of CAF; TS, test session; CMJ, countermovement jump test; MATT, modified agility *t*-test; RSA, repeated sprint ability test; RPE, rating of perceived exertion; QUEST, questionnaire on caffeine adverse side effects.

A double-blind, placebo-controlled, randomized crossover design was used in this investigation, where each player acted as her own control. Players performed four different experimental sessions with at least a 72 h washout period between each session to allow complete recovery and substance washout. Throughout the four experimental trials, the subjects were administered either a placebo (PLAC), 3 mg·kg^−1^ CAF (CAF-3), 6 mg·kg^−1^ CAF (CAF-6), or 9 mg·kg^−1^ CAF (CAF-9). An independent investigator who was not part of the data collection process facilitated the randomization, blinding, and supplement preparation (Figure 1).

Over four weeks before the experiment, participants visited the facility numerous times for familiarization and data assessment. The initial visit involved explaining the study procedures, obtaining informed consent, assessing anthropometric data, and completing all the questionnaires (daily caloric intake, PSQI, MEQ, and habitual CAF intake). The body mass of each participant was recorded with minimal clothing, no shoes, and an empty stomach using the digital scale Tanita BC-545n (Tanita Corporation, Arlington Heights, IL, USA) to the nearest 0.1 kg. After identifying the appropriate CAF dose for each participant, they were asked to indicate their dietary intake before the first experimental day and were asked to follow the same diet during the next visits. During the two visits leading up to the study, all players were familiarized with the experimental protocol and the equipment at the same time as the experimental trials (09:00 AM) to reduce learning effects during the experiment and guarantee high-quality results.

During each session of the four testing trials, the athletes underwent a standardized warm-up (~10 min including rest) that began 60 min after CAF ingestion. This warm-up included 3 min of jogging (at 8–10 km/h), followed by 3 min of whole-body dynamic stretching. The stretching was then succeeded by 2 min of sprinting (ankling, high-knee, back kicking, and skipping) and 2 min of sprinting. Participants were then tested during each session in the same order: countermovement jumps test (CMJ), modified agility t-test (MATT), repeated sprint ability (RSA) test, rating of perceived exertion (RPE) test, and, lastly, the CAF side effects’ questionnaire. A resting interval of 5 min was taken between each of these tests to ensure proper recovery.

To maintain uniformity in the measurements, all tests were conducted in an identical indoor court utilizing the same testing equipment supervised by the same investigators and following the same sequence. The testing was carried out in an indoor court with similar ambient temperatures (approximately 26 °C) and relative humidity (49%) throughout the four experimental sessions of our investigation. Participants were asked to adhere to the following instructions 24 h prior to each trial: (i) to avoid any medications or dietary supplements and not to use any performance-enhancing substances for the duration of the study or two weeks prior to participating; (ii) to continue regular training but avoid intense workouts before the day prior to each trial; (iii) to eat and drink the same as usual; (iv) to abstain from stimulants and CAF, for which participants were given a list of food and drink items containing CAF and were requested to avoid consuming these items for a full day prior to each exercise testing session; and (v) to ensure a minimum of 7 h of sleep the night before the experiment. To ensure uniformity across the trials, participants were asked to maintain a 24 h record of their habits before the initial trial. They were then instructed to replicate these same habits before undertaking the second, third, and fourth trials.

### 2.3. CAF Administration Protocol

After two familiarization sessions, the experiment was carried out at the same time across four trials (at 09:00 AM) to minimize the impact of circadian rhythm disturbances, following a double placebo method for CAF and PLAC content. Unidentifiable capsules containing either an appropriate dose of CAF (Bulk Powders, Unit 1, Gunfleet Business Park, Brunel Way, Highwoods, Colchester CO4 9QX, UK) or an inert compound (Cellulose; Guinama 6, Spain) as PLAC were consumed by the participants in each condition. To allow for absorption, the capsule was ingested with 10 µL of water one hour before the trial began and at least 1 h after the last meal to maintain the same duration of absorption. In each experiment, the substances under scrutiny were contained within opaque capsules without taste or smell consumed 60 min before the test was started to allow for optimal CAF absorption. Since participants arrived at the indoor training court following at least seven hours of fasting and to reduce potential gastrointestinal distress, a standardized breakfast consisting of approximately 450 kcal was administered 120–150 min before each session. This routine was a shift from their usual practice during trial days, where they would typically arrive at the court a couple of hours early.

### 2.4. Habitual CAF Intake Assessment

The method of quantifying regular CAF intake was adapted from the Food Frequency Questionnaire (FFQ) as initially suggested by Bühler et al. [51]. Measurements were based on household quantities to determine the individual’s daily food consumption over four weeks before the commencement of the experiment, adhering to earlier suggestions [50].

An experienced nutritionist utilized nutritional tables for database construction to calculate the daily CAF intake for each athlete during the four weeks leading up to the experimental trials, considering the body mass of each athlete as suggested by Filip et al. [50]. According to the classification by Filip et al. [50], individuals with daily CAF consumption between 25 mg·day^−1^ and 0.99 mg·kg·day^−1^ were considered low consumers, while those with consumption between 1.00–2.99 mg·kg·day^−1^ were considered mild consumers. All our participants were mild consumers of CAF, with an average intake of 1.09 ± 0.3 mg·kg·day^−1^.

### 2.5. CMJ

Participants were directed to perform a quick vertical jump, which involved a downward preparatory movement and an upward propulsive phase, all while maintaining an upright stance [55,56]. The participants were guided to aim for maximum height during each jump, ensuring that they landed in the original position, with their hands placed on their hips to limit the influence of arm swings. Both the Optojump-next equipment (Bolzano, Italy) and the accompanying Microgate software v.1.13.24 (Optojump software, version 1.10.50) were utilized in this process. The only data taken into account for the analysis were the height of the jump, measured in centimeters. Each participant carried out three trials, with a two-minute rest period between each trial. The highest jump achieved within these three attempts was kept for our analysis.

### 2.6. Modified Agility t-Test (MATT)

The agility of the participants was assessed using a modified t-test, which involved navigating a course marked by four cones placed in a “t” shape [57]. The modified agility *t*-test involved multidirectional sprinting, shuffling, and backpedaling. A timing gate (Witty, Microgate^®^, Bolzano, Italy) was used to record the time at the start/finish line (the same line used for this test), while the athletes began the test 0.5 m behind the gate. Participants began with a 5 m linear sprint to cone B, followed by a 2.5 m leftward shuffle to cone C, a 5 m rightward shuffle to cone D, a 2.5 m leftward shuffle back to cone B, and a 5 m linear backpedal to cone A. The lengths between B–C and B–D were set at 2.5 m, while A–B spanned 5 m. For each trial, the total distance covered was 20 m. The MATT performance score was the fastest time from two trials, interspersed with three minutes of rest.

### 2.7. RSA Test

In line with prior studies on female athletes [58,59,60], the repeated sprint ability (RSA) test was conducted. The test involved six maximal 2 × 12.5 m shuttle sprints, each separated by 20 s of passive rest and 180° turns. The RSA was designed to evaluate repeated-sprint performance with directional changes. The timing gates (Witty, Microgate^®^, Italy) recorded the timings. Athletes received verbal encouragement during the test and were directed to position themselves 0.5 m behind the starting line, 6 s before the start of each sprint until the subsequent start signal. Disruption of the photocell beam led to the automatic initiation of the digital timer. A light panel (Microgate^®^, Italy) was used to provide a visual countdown of 3 s. Athletes sprinted 12.5 m from the starting line, touched the second line with their foot, and then returned to the starting line as quickly as possible. Participants were instructed to complete all sprints at their fastest speed. For further analysis, the fastest sprint (RSA_best_) and the average time of all sprints (RSA_mean_) were recorded.

### 2.8. Rating of Perceived Exertion (RPE)

After every 25 m shuttle sprint of the RSA test, the RPE was verbally assessed. The mean of these six RPE measurements was subsequently used as the final score. According to the Borg [61,62] scale of 10 points, a score of 0 indicates a state of rest, similar to sitting in a chair, whereas a score of 10 suggests engagement in extremely strenuous physical activity.

### 2.9. CAF Adverse Side Effects Questionnaire

To evaluate potential adverse effects such as headaches, sleep disturbances, or gastrointestinal problems that could be linked to CAF intake, participants were instructed to complete a questionnaire. This nine-item measure employs a dichotomous (yes/no) response scale for CAF ingestion, as outlined by Pallares et al. [63]. This questionnaire was administered immediately after each experimental trial (QUEST + 0 h) and again the following day (QUEST + 24 h).

### 2.10. Statistical Analysis

STATISTICA 10 software (StatSoft, Paris, France) was used for all the statistical analyses. The mean and standard deviation (SD) of each variable were determined. Figures were created using GraphPad Prism 8 (GraphPad Software, San Diego, CA, USA). Parametric tests were implemented when the Shapiro–Wilk test confirmed a normal data distribution. The impact of different doses of CAF was examined using one-way ANOVA (four conditions) with repeated measures. When appropriate, Tukey’s HSD post hoc test was performed to test for significant differences between means. A *p*-value ≤ 0.05 was considered to indicate statistical significance. Moreover, effect sizes (Cohen’s d) were calculated using partial eta squared (ηp^2^). Parametric effect sizes were defined as large (≥0.14), moderate (≥0.06), or small (≥0.01) [64]. Furthermore, the chi-square test was used to examine variations in the occurrence of different CAF dose side effects in athletes during the same trial day and 24 h later.

## 3. Results

### 3.1. CMJ

**Figure 2:**
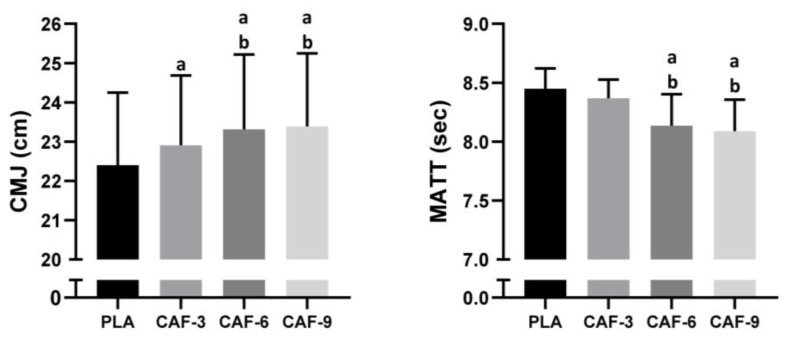
Mean ± SD values of the countermovement jump test (CMJ) and modified agility t-test (MATT) results observed under four conditions: PLA, placebo; CAF-3, 3 mg·kg^−1^ CAF; CAF-6, 6 mg·kg^−1^ CAF; CAF-9, 9 mg·kg^−1^ CAF. ^a^ (*p* < 0.05), significant difference compared to the placebo group; ^b^ (*p* < 0.05), significant difference compared to CAF-3 condition.

A statistical analysis of CMJ revealed a significant main effect of CAF intake (F (3, 45) = 25.79; *p* < 0.001; ηp^2^ = 0.63). Compared to that of the PLAC, the CMJ performance was better after treatment with CAF-3 (2.3%, *p* < 0.01), CAF-6 (4.1%, *p* < 0.001), or CAF-9 (4.4%, *p* < 0.001). Tukey’s post hoc test revealed significantly lower CMJ performance after CAF-3 ingestion than after CAF-6 (*p* < 0.05) or CAF-9 (*p* < 0.01) ingestion. However, there was no significant difference in CMJ between the CAF-6 and CAF-9 conditions (*p* > 0.05) (Figure 2).

### 3.2. MATT

There was a significant main effect of CAF supplementation on agility performance (F (3, 45) = 18.61; *p* < 0.001; ηp^2^ = 0.55). One-way ANOVA indicated a statistically significant difference in the CAF-6 (−3.7%, *p* < 0.001) and CAF-9 (−4.3%, *p* < 0.001) conditions compared to the PLAC condition. However, there was no significant difference in CMJ between the PLAC and CAF-3 groups (*p* > 0.05). Compared to those of CAF-3, agility performance was better under the CAF-6 (*p* < 0.01) and CAF-9 (*p* < 0.001) conditions. Furthermore, there was no significant difference in the MATT between the CAF-6 and CAF-9 conditions (*p* > 0.05) (Figure 2).

### 3.3. RSA Test

#### 3.3.1. RSA_mean_

**Figure 3:**
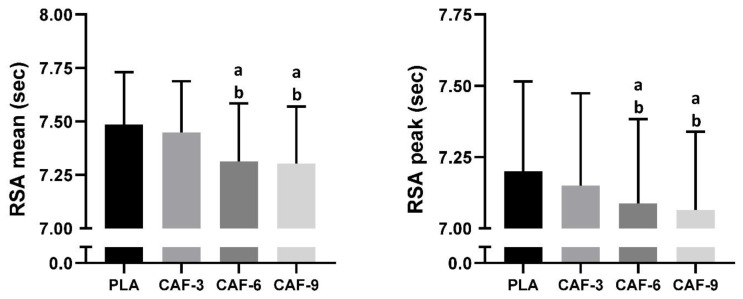
Mean ± SD values of repeated sprint ability (RSA) performance measured under four conditions: PLA, placebo; CAF-3, 3 mg.kg^−1^ of CAF; CAF-6, 6 mg·kg^−1^ of CAF; CAF-9, 9 mg·kg^−1^ of CAF. ^a^ (*p* < 0.05), significant difference compared to the placebo group; ^b^ (*p* < 0.05), significant difference compared to CAF-3 condition.

The RSA_mean_ indicated significant main effects of pre-exercise CAF intake (F (3, 45) = 67.89; *p* < 0.001; ηp^2^ = 0.81). Tukey’s post hoc test revealed that the mean RSA volume was better only in the CAF-6 (−2.3%, *p* < 0.001) and CAF-9 (−2.4%, *p* < 0.001) conditions than in the PLAC condition. Compared to that of CAF-3, the mean RSA was greater in the CAF-6 (*p* < 0.001) and CAF-9 (*p* < 0.001) conditions. However, no significant difference between the CAF-6 and CAF-9 (*p* > 0.05) conditions was observed (Figure 3).

#### 3.3.2. RSA_best_

Statistical analysis of RSA _Best_ showed a significant main effect of CAF intake (F (3, 45) = 17.13; *p* < 0.001; ηp^2^ = 0.53). In comparison to the placebo, CAF intake enhanced RSA_best_ only under the CAF-6 (−1.6%, *p* < 0.001) and CAF-9 (−1.9%, *p* < 0.001) conditions. The RSA_best_ was better in the CAF-6 (*p* < 0.05) and CAF-9 (*p* < 0.01) conditions than in the CAF-3 condition. However, no significant difference between the CAF-6 and CAF-9 (*p* > 0.05) conditions was found (Figure 3).

### 3.4. RPE

**Figure 4:**
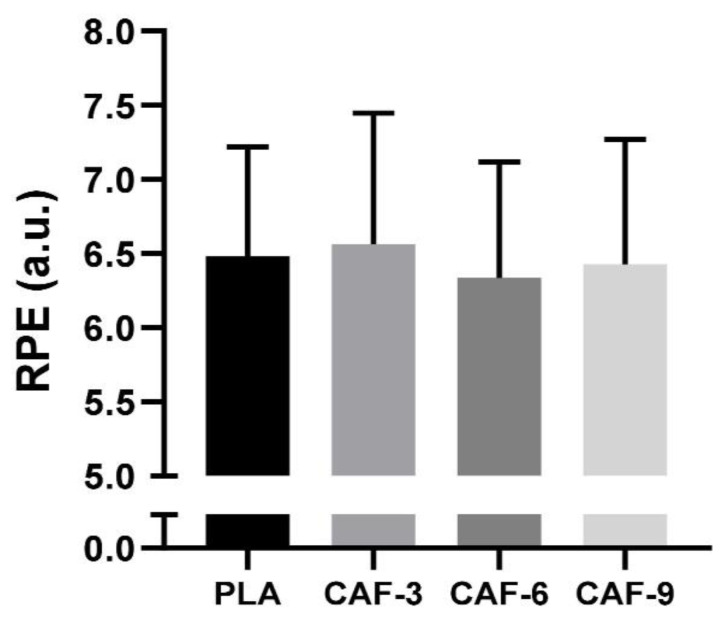
Mean ± SD values of the rating of perceived exertion (RPE) recorded under four conditions: PLA, placebo; CAF-3, 3 mg·kg^−1^ CAF; CAF-6, 6 mg·kg^−1^ CAF; CAF-9, 9 mg·kg^−1^ CAF.

There were no significant effects of different doses of CAF intake on RPE scores (F (3, 45) = 1.87; *p* = 0.14; ηp^2^ = 0.11) (Figure 4).

### 3.5. Adverse Side Effects Questionnaire

Immediately after the PLAC trials, our results revealed a low frequency of side effects (0–18.75%; QUEST + 0 h). Regarding the different CAF dose effects, athletes indicated a low frequency of side effects such as muscle soreness and gastrointestinal problems under the CAF-3 (0–18.75%; QUEST + 0 h) and CAF-6 (0–18.75%; QUEST + 0 h) conditions. However, a moderate frequency of side effects such as tachycardia, heart palpitations, and gastrointestinal problems was observed after the CAF-9 trial (6.25–25%; QUEST + 0 h). Twenty-four hours after each testing trial (QUEST + 24 h), the side effects were low and similar under the conditions of PLAC (0–6.25%; QUEST + 0 h), CAF-3 (0–6.25%; QUEST + 0 h), and CAF-6 (0–12.5%; QUEST + 0 h). However, 24 h after CAF-9 ingestion (12.5–31.25%; QUEST + 0 h), more athletes reported increased urine output (31.25%), gastrointestinal problems (31.25%), increased vigor/activeness (25%), headache (25%), and insomnia (25%) (Table 2). We found that different CAF doses led to gastrointestinal problems (x^2^ = 16.27, *p* = 0.001) and an increase in vigor/activeness (x^2^ = 9.33, *p* = 0.025) at (QUEST + 24 h), with higher side effects observed following the ingestion of CAF-9. However, no significant effect of different CAF doses was reported for all other side effects (all *p* > 0.05). Despite this, no athlete (0/16) recognized the correct complete experimental trials, suggesting that blinding was effective.

## 4. Discussion

The main objective of this study was to investigate the effect of three different doses of CAF intake on short-term maximal performance among young female athletes. The key outcomes of this investigation indicate that irrespective of the consumed dose, CAF significantly enhanced CMJ performance but not RPE score. However, a noticeable improvement in agility and RSA performance was observed after the administration of 6 or 9 mg·kg^−1^ of CAF, whereas no such improvement was observed after the ingestion of 3 mg·kg^−1^. Furthermore, increasing CAF intake from 6 to 9 mg·kg^−1^ of CAF did not result in a significant enhancement of performance among mild CAF consumers in short-term, high-intensity exercise. However, this higher dosage (9 mg·kg^−1^) was associated with an increase in the frequency of adverse side effects.

Recent findings have indicated that a specific range of CAF doses varying from 3–6 mg·kg^−1^ enhances performance in team sports [16]. The 3 mg·kg^−1^ and 6 mg·kg^−1^ doses were selected based on their observed impacts on muscle performance during team-sports games and their ergogenic effects on short-term maximal performance in female athletes [15,16]. We opted for these CAF dosages because 3 mg·kg^−1^ represents the minimum dose that influences muscle performance during team-sports exercise, while 6 mg·kg^−1^ represents a higher dose known to demonstrate ergogenic effects on short-term maximal performance in female athletes. While previous data have demonstrated that a higher dosage of CAF such as 9 mg·kg^−1^ enhances endurance [65,66], its impact on short-term maximal performance in female athletes has not been specifically assessed.

Our findings demonstrated that, compared to the placebo, the three varying doses of CAF significantly improved CMJ performance, with better performance observed after 6 and 9 mg·kg^−1^. This finding aligns with that of a recent meta-analysis conducted on female team sports [15]. Regarding the impact of different CAF dosages on CMJ performance, it was observed that a lower dose of CAF (3 mg·kg^−1^ or less) significantly enhanced CMJ height in female low-CAF consumers [30]. Similarly, this dosage, when consumed through a caffeinated drink, appeared to improve the CMJ performance of female volleyball players [26]. A moderate dose of CAF (6 mg·kg^−1^), when administered in capsule form, did not enhance CMJ in team-sports samples [27]. However, the same dosage but in capsule form seemed to improve CMJ performance in female football players who are low-CAF consumers [33]. Furthermore, this specific CAF dosage and form of administration appeared to enhance squat jump performance in female handball players [32]. However, studies investigating the effects of higher doses such as 9 mg·kg^−1^ on female team-sports are lacking, making it challenging to compare and derive conclusions from our results.

Regarding agility performance, which is crucial in these sports, our results suggest that CAF supplementation yields effective results following the ingestion of either 6 or 9 mg·kg^−1^; however, there was no significant effect observed with a 3 mg·kg^−1^ dosage. Our findings align with a recent meta-analysis that indicated the effectiveness of CAF supplementation in enhancing agility performance [17]. However, these findings significantly contrast with those of another meta-analysis that found no significant effect of CAF intake on agility performance among female team-sports athletes [15].

Regarding the effects of pre-exercise CAF on agility performance in female team-sports athletes, lower doses of less than 3 mg·kg^−1^ (1.39 mg·kg^−1^) did not seem to enhance agility performance in the fatigued state in female volleyball players when using a power gel [22]. A similar dose of CAF (1.3 mg·kg^−1^) in combination with a caffeinated drink did not enhance agility in female football players in the resting state but did enhance agility in the fatigued state [24]. A dose of 3 mg·kg^−1^ of a caffeinated drink enhanced agility in the resting state among female volleyball players [26]. However, using the same dose of CAF in capsules did not enhance female agility performance in low-CAF-consumer basketball players [23] or handball players [30]. A moderate dose (6 mg·kg^−1^) of CAF administered via capsules did not affect the performance of agility tests in female basketball or volleyball players [29]. A recent study showed that administering a dosage of (5 mg·kg^−1^) of CAF can enhance CMJ and grip in female volleyball players throughout a week-long training period [34].

However, employing the same protocol resulted in improved performance in the agility test for female handball players [32]. The observed variance in the data might be attributed to differences in the dosage and form of CAF supplementation as well as whether the trials were conducted under rested or fatigued conditions. Furthermore, an individual’s daily habitual CAF consumption and the respective correlation with appropriate doses may also contribute to the observed divergence in results. Notably, according to the recent classification system of Filip et al. [50], daily lower-CAF consumers (between 25 mg·day^−1^ and 99 mg·kg.day^−1^) are not classified as naive consumers (less than 25 mg·day^−1^) or mild consumers (between 1.00 and 2.99 mg·kg^−1^), which could impact the interpretation of the results. Additionally, the disparity observed in results across studies could be attributed to the varying menstrual cycle phases during which trials were conducted, which may interact with physical performance and the body’s response to CAF. It is was shown that women experience hormonal fluctuations due to the menstrual cycle that can impact athletic performance [67,68]. For example, evidence suggests that different phases of the menstrual cycle can affect strength performances [69]. Additionally, the consumption of oral contraceptives can alter CAF metabolism, leading to an extended half-life and sustained effects in the body [70]. Our study deliberately excluded female athletes who use any form of contraception to maintain homogeneity in the results; however, few studies consider such variables [18]. Although previous investigations point to the similarity of bodily absorption and effectiveness of acute CAF intake across various menstrual phases in female athletes [53], some studies hint at a possible reduction in CAF’s ergogenic benefits due to its interaction with female sex hormones [71].

Regarding the mechanism responsible for the observed effectiveness, the data indicate that CAF has the potential to enhance anaerobic exercise, although the underlying reasons remain elusive [18]. Recognized for triggering an increase in Ca^2+^ and K^+^ levels, CAF promotes the development of cross-bridges by raising Ca^2+^ from the sarcoplasmic reticulum, thereby augmenting muscle power [39]. Concurrently, the increase in the serum K^+^ concentration results in a surge in Na+/K+ ATPase activity, which may help mitigate muscle fatigue [72]. Therefore, while there is evidence that CAF could enhance female running speed and agility in team-sports tests, further investigations are needed to confirm whether this influence extends to sprint actions during real matches.

Given that team sports include multiple sprints with intermittent, low-intensity periods, we carried out the RSA test to assess female athletes’ ability to sustain sprint intensity. Our study found that consuming caffeine in either 6 or 9 mg·kg^−1^ doses before exercise improves both peak and mean outcomes in the RSA test compared to a placebo. No significant difference was detected when a dosage of 3 mg·kg^−1^ was administered. These findings corroborate certain existing systematic reviews and meta-analyses that involved male and female participants in team sports [17,18]. Nevertheless, they diverge from the conclusions of a recent systematic review [19]. The disparity in these results could primarily be attributed to the small number of included studies investigating this specific performance aspect in female athletes.

Regarding repeated sprint performance, a lower dose (1.39 mg·kg^−1^) of a power gel did not enhance this performance among female volleyball players [22]. For female rugby players with low caffeine consumption, the administration of powdered caffeine (3 mg·kg^−1^) did not improve repeated sprinting performance; however, it enhanced the total covered distance [25]. Using the same dose administered in capsule form, Stojanovic et al. [23] did not observe improvements in repeated sprinting performance among low-CAF-consumer basketball players. A moderate dose of CAF (6 mg·kg^−1^) in a capsule form enhanced the number of repeated sprints and total distance covered among female football players [33] and the peak distance and total distance traveled during the 5 m shuttle test on young female handball players [32]. However, using the same protocol of CAF supplementation did not improve sprint ability in basketball or football players [31]. Reflecting on the time interval required between placebo and CAF trials, prior studies in female team sports have implemented washout periods ranging from 48 to 96 h [1,24,36]. In our study, we decided on a minimum washout duration of 72 h following previous studies in female team sports [24,25,73,74]. This interval was selected also to ensure the necessary recovery time between trial sessions in CAF experimental protocol, which is in alignment with previous studies including repeated sprint bouts for female team-sport athletes such as the RSA test [25] and the 5 m shuttle test [75].

The inconclusive nature of the existing data presents a challenging scenario regarding the optimal dosage of CAF, which does not seem to generally fall within the range of 3 to 6 mg·kg^−1^ for all individuals. In light of these findings, future research should take into consideration the effects of lower CAF doses (≤3 mg·kg^−1^), primarily on the central nervous system (CNS) [38], while considering that an increase in dosage (i.e., 6–9 mg·kg^−1^) might trigger peripheral effects [8,10]. We suggest that the intricacies of the prescribed exercise, which involve multifaceted executive aspects such as directional changes during sprinting, may necessitate a higher dosage for optimal enhancement in transitioning from central to peripheral execution. Nevertheless, it appears that the relationship between the CAF dosage and erogeneity follows a linear pattern only up to the point at which the optimal dose is reached; beyond this threshold, further increases in dosage do not lead to improved performance. It is therefore crucial that the effects of higher CAF dosages be explored in the context of the individual’s tolerance and sensitivity to this substance. Furthermore, genetic factors as well as daily habits should be factored into these considerations. Studies suggest that frequent CAF intake may increase adenosine receptor levels in brain tissue [76,77]. This finding implies that regular and occasional CAF users might have different reactions to CAF intake during physical activity. Moreover, the literature has yet to clearly determine the shortest duration of CAF abstinence in one’s diet that would effectively enhance sensitivity to its effects [39]. Based on these observations, it is plausible to hypothesize that there may be a correlation between habitual CAF consumption, dosage and abstention period before experimentation, and sensitivity to the impact of CAF on physical performance. This complex relationship warrants further investigation to fully comprehend its dynamics and implications. In doing so, we may be in a position to establish an optimal protocol for CAF administration that effectively enhances overall female athlete performance.

On the other hand, our study’s results revealed that RPE scores did not significantly differ from those of the placebo group. Additionally, these scores were not influenced by CAF supplementation irrespective of the three varying doses of CAF ingested. This finding aligns with those of meta-analyses [17,78] showing minimal improvements in performance aspects such as jumping, sprinting, agility, and team-sport-specific endurance with moderate CAF consumption (3–6 mg·kg^−1^), with no change in perceived exertion during physical exercise. These results are further supported by a recent meta-analysis on women’s team sports [15].

Regarding ratings of RPE scores on female team-sports athletes, a study by Astorino et al. [24] revealed that a lower dose of CAF (1.3 mg·kg^−1^) increased RPE scores among female football players who were low consumers of CAF. As previously discussed by Bougrine et al. [32], our study suggested that RPE scores remained consistent due to specific temporal factors involved in the RSA test. A brief recovery period of 20 s between sprints does not offer sufficient time for CAF to overcome the fatigue that accumulates during a repeated sprint test. This could explain why CAF did not demonstrate a significant impact on reducing the participants’ perceived exertion. Furthermore, the observed outcomes imply that mechanisms beyond a decrease in RPE may account for the effectiveness of CAF. The ergogenic effects of the CAF, as demonstrated in this study, could be attributed to its potential to accelerate muscle fiber conduction velocity and intensify motor unit recruitment [79,80]. It is crucial to recognize the potential influence of the diverse performance tests utilized on the perceived effect of CAF on the RPE. In fact, the order in which these tests were conducted may have influenced the results; for instance, initiating the session with a CMJ and agility test could have altered the RPE responses in the subsequent RSA test.

Finally, establishing the ergogenic efficacy of low CAF doses in comparison to higher doses presents significant challenges when examining existing studies. This difficulty arises owing to the substantial variability observed in CAF-induced performance enhancement across various trials and participants. As a result, there is a pressing need for additional research investigating the effects of varying CAF doses within the same individuals, thereby allowing for direct comparisons and more robust conclusions. The ergogenic effects of CAF on athletic performance can be modulated by a multitude of factors. These include but are not limited to genotype [14,81], training status [82], habitual CAF consumption [83], sex [84], CAF origin and form [85], and age [86]. Factors such as the duration of CAF abstinence prior to experimentation, potential side effects [37], the nocebo effect [87], the type of exercise [88], and the timing of CAF administration [32,89] also play significant roles in its ergogenic modulation. In our study, CAF was ingested 60 minutes before trials. This timing correlates with the peak plasma CAF concentration, which is typically achieved between 15 and 120 minutes following oral ingestion [90]. CAF is known for its rapid absorption into the bloodstream via the gastrointestinal tract, where it reaches a peak plasma concentration approximately 60 minutes after ingestion [16]. These diverse factors, each with a potential impact on the outcomes, create a significant level of variance in the methodologies and sample populations of existing studies. Considering the underrepresentation of female athletes in most studies examining these factors, a distinct comparative approach may be necessary to gain clearer insights into the ergogenic effects of CAF. Regarding age, recent findings suggest that CAF’s performance-boosting effects may vary with age due to changes in muscle growth and degradation [86]. Interestingly, CAF appears to offer significant athletic benefits for older individuals, potentially aiding in daily functional activities [86]. However, there are limited studies focused on younger female athletes. Insights from a recent meta-analysis that examined the impact of CAF on female team-sport athletes highlighted contributions from 18 studies involving a total of 240 young-adult female athletes [15]. These studies presented a mean age ranging from 18 to 26 years. To the best of our knowledge, only a recent study included female athletes with a mean age of 16.3 ± 0.8 years [32]. The findings of our study align with the results of Bougrine et al. [32] and suggest that a moderate dose of CAF intake can be beneficial in enhancing vertical jump, agility, and 5 m shuttle sprint performance among young female handball players. Limited studies have explored CAF’s impact on young athletes, resulting in a scarcity of evidence that draws definitive conclusions or guidelines. The performance advantages seen in adults may not extend to younger individuals, highlighting the need for additional research in the youth athlete demographic [16].

Regarding the undesirable side effects resulting from CAF consumption, our findings suggest a notable relationship correlation with dosage. More specifically, higher incidences of these side effects were recorded 24 h after the ingestion of 9 mg·kg^−1^ of CAF. Conversely, fewer instances were observed following the consumption of a smaller (3 mg·kg^−1^) dosage. These data underscore the potential role of CAF dosage in mediating its side effects. There is a noticeable lack of data concerning the potential side effects of the typical amounts of CAF consumed to enhance athletic performance, which typically range from 1 to 9 mg·kg^−1^ [89]. Our results support a previous study where it was determined that consuming CAF in the morning, specifically at a dose of 9 mg·kg^−1^, frequently resulted in adverse effects [63]. These included tachycardia, anxiety, gastrointestinal problems, and notably, sleep disturbances, affecting 35–40% of participants [63]. Nevertheless, the impact of 6 mg·kg^−1^ CAF side effects was more noticeable in the day following its administration, particularly when consumed in the afternoon rather than in the morning [32,89]. Interestingly, studies have yet to measure the performance-enhancing effects of CAF throughout varying stages of the menstrual cycle, even though CAF and female sex hormones such as estrogen are known to interact [91]. This could be the underlying mechanism that leads to an increase in side effects following the ingestion of high doses of CAF (9 mg·kg^−1^) in our current study. Regarding the influence of estrogens on muscle function, while certain studies have indicated an enhancement in muscle contractility due to a shift from low- to high-force cross-bridge transitions in muscles [92,93], the overall consensus is that muscle strength remains consistent throughout the phases of an ovulatory menstrual cycle [94]. Notably, there is evidence suggesting that caffeine’s impact on elevating blood pressure is more pronounced during the follicular phase than in the luteal phase among young females [95]. In this context, all studies on CAF highlight the withdrawal effects triggered by avoiding intake before performance tests, although the cessation period varies across studies, ranging between 6 h and 10 days [37]. However, the surprisingly significant lack of research on the effects of CAF withdrawal on exercise performance is critical, especially when developing an effective restriction protocol. Notably, CAF withdrawal symptoms typically appear between 12–48 h after the last intake, leading to various clinical outcomes irrespective of habitual CAF consumption [96]. Given that the half-life of CAF can range from 3 to 7 h, it could interfere with the initial state of participants in studies involving brief periods of CAF deprivation less than 24 h [97].

The nocebo effect could influence the prevalence of undesirable side effects related to reducing CAF intake among regular consumers [98]. However, the relationship between reduced CAF intake and potential impacts on physical performance remains uncertain. It is unclear whether the alleviation of withdrawal symptoms achieved through an effectively blinded sample could influence exercise performance. Particularly in the case of female athletes who compete multiple times in short periods, it is crucial to understand whether performance enhancement might inadvertently lead to adverse side effects. Interestingly, while the positive effects of CAF on physical factors are acknowledged, caution is advised for team-sport athletes because its consumption may negatively affect technical and tactical aspects, potentially leading to a decrease in accuracy performance due to associated side effects [16]. However, further research is needed to elucidate these connections and ensure that performance improvements do not come at the expense of athletes’ health.

### Limitations

The current investigation has several limitations that should be acknowledged. A potential shortcoming of our study is the lack of measurements of blood CAF levels, which makes the actual absorption impact of various CAF dosages unclear. Blood variable data were not collected in this study, thus precluding an investigation into the potential metabolic and hormonal influences on physical performance. It should be noted that our study did not examine the influence of the menstrual cycle on performance and caffeine gains. Although it has been suggested that the interaction between caffeine and female sex hormones may reduce the performance-enhancing benefits of caffeine [71], this interaction was not within the scope of our study. Further studies are needed to fully understand this interaction and its implications for female athletes. Before the initial experiment, participants’ diets, particularly their avoidance of CAF-rich foods, were recorded. This dietary regimen was repeated in later sessions. The collection of urine samples helped verify that all participants excluded CAF from their diets. The current study, which was conducted on young female athletes, restricts the direct applicability of the findings to other demographic groups such as males, inactive individuals, older people, and heavy consumers of CAF. Notably, differences in individuals’ reactions to CAF are often linked to variations in the CYP1A2 gene. This gene influences the metabolism of CAF; individuals with the AA genotype seem to derive more exercise benefits than those with the AC/CC genotype [99]. Regrettably, our study did not collect data on these genetic variations, which provides an avenue for consideration in future research.

## 5. Conclusions

In conclusion, the study established that the intake of 3 mg·kg^−1^ CAF 60 min before testing significantly enhanced jumping performance in female team-sports players who were mild CAF consumers. Nonetheless, this CAF dose did not have any considerable impact on agility or repeated sprint bouts among the same population. Remarkably, doses of 6 and 9 mg·kg^−1^ enhanced all these parameters. Our findings revealed that for short-term high-intensity exercise, there was no significant increase in performance among mild CAF consumers when the intake of CAF was increased from 6 to 9 mg·kg^−1^; however, this higher dosage was associated with a notable increase in the frequency of adverse side effects.

Therefore, the findings highlight the recommendation of consuming a moderate dose of 6 mg·kg^−1^ of CAF as opposed to 3 or 9 mg·kg^−1^ to optimize various aspects of short-term maximal performance in young female team-sports players without the prevalence of disturbing CAF side effects. Athletes and coaches may find this recommendation useful in implementing caffeine intake strategies throughout busy training and tournament schedules.

## Figures and Tables

**Table 1:** General characteristics of the study subjects (n = 16).

	Minimum	Maximum	Mean
**Age** (years)	16	18	16.9 ± 0.6
**Body mass** (kg)	50.3	74.1	60.1 ± 5.7
**Height** (m)	1.6	1.8	1.64 ± 0.1
**Body mass index** (kg·m^−2^)	18.3	23.3	21.6 ± 1.5
**CAF habitual intake** (Mg·kg·day^−1^)	0.99	1.52	1.09 ± 0.3
**Sleep duration** (h)	6.9	9.1	7.7 ± 0.6
**MEQ questionnaire score** (au)	44	58	48.9 ± 4.5
**Practice experience** (years)	4	7	5.1 ± 0.9
**Training sessions frequency/week** (au)	3	5	4.3 ± 0.7
**Daily caloric intake** (Kcal)	1650	2996	2335.8 ± 404.4
**Menstrual cycle length** (days)	25	31	27.69 ± 1.93

MEQ, Morningness–Eveningness Questionnaire of Horne and Ostberg (1976). The minimum, maximum, mean, and standard deviation values of the participants’ characteristics are shown in the table.

**Table 2:** Side effects were reported by participants immediately after the end of each experimental trial (QUEST + 0 h) and after 24 h (QUEST + 24 h). The data are presented as the percentage of individuals with affirmative responses to each of the side effects obtained from 16 players.

	PLAC		CAF-3		CAF-6		CAF-9	
	+0 h	+24 h	+0 h	+24 h	+0 h	+24 h	+0 h	+24 h
Muscle soreness (%)	6.25	6.25	0	6.25	6.25	6.25	6.25	12.5
Increased urine output (%)	0	6.25	0	6.25	0	12.5	6.25	31.25
Tachycardia and heart palpitations (%)	0	0	12.5	6.25	12.5	6.25	25	25
Anxiety or nervousness (%)	0	0	0	0	0	6.25	6.25	18.75
Headache (%)	0	0	0	6.25	6.25	12.5	12.5	25
Gastrointestinal problems (%)	6.25	0	6.25	0	12.5	0	12.5	31.25
Insomnia (%)	-	6.25	-	0	-	6.25	-	25
Increased vigor/activeness (%)	6.25	0	6.25	0	0	6.25	12.5	25
Perception of performance improvement (%)	18.75	-	18.75	-	18.75	-	12.5	-

## Data Availability

Data are available upon reasonable request from the first author.
